# Patient age as risk factor for revision of ventriculopleural shunt

**DOI:** 10.1186/2045-8118-12-S1-P34

**Published:** 2015-09-18

**Authors:** Edward Frederick Melamed, Parham Yashar, Eisha Anne Christian, Cherisse Berry, J Gordon McComb

**Affiliations:** 1 Division of Neurosurgery, Children's Hospital Los Angeles, CA, USA; 2 Department of Neurosurgery, Keck School of Medicine, Univeristy of Southern California, Los Angeles, CA, USA

## Introduction

Ventriculoperitoneal shunts remain the standard of care for the treatment of hydrocephalus. However, for patients at risk of abdominal infection (e.g. following appendiceal rupture), those with a recent history of multiple abdominal surgeries, or for those who experience persistent symptoms due to inadequate absorption and possible pseudocyst, the ventriculopleural shunt (VPL) is a viable alternative.

## Methods

We identified pediatric patients who underwent their initial VPL insertion at our institution from 1977-2013. Data was collected retrospectively from the clinical charts.

## Results

131 (78 M) patients were identified. Mean age at insertion of ventriculopleural shunt was 14 ± 5 years. Follow up was available on 124 patients with a mean duration of 50 months (range 0.1-288). Prior to and including VPL insertion, 58 patients with available preoperative data had experienced a mean of 3 ± 3 revisions. These patients underwent a mean of 1 ± 1 subsequent revisions of their VPL, significantly fewer than those of their prior shunt (p < 0.01).

At sixteen months post VPL insertion, half of the 102 patients with at least one year of follow up had undergone a shunt reoperation, either a revision or conversion to another shunt. 18/34 patients (53%) between twelve to sixteen-years-of-age and 21/39 (46%) patients aged seventeen or older required at least one subsequent VPL change in contrast to 23/29 (79%) patients under twelve-years-old at the time of insertion (p < 0.05). Kaplan Meir survival curves for the three age groups deviated significantly (p = 0.01). Furthermore, the hazard ratio of undergoing a shunt reoperation for patients under twelve was 1.9 (95% CI 1.2, 3.0) in comparison to either of the two older patient groups.

## Conclusion

The ventriculopleural shunt is a viable alternative for patients who cannot tolerate a VP shunt or experience persistent symptoms. After insertion these patients may be less likely to require additional revisions. The patient's age should be among the factors surgeons weigh carefully prior to VPL insertion. In comparison to older adolescents, children under twelve who undergo conversion to VPL may be at greater risk for subsequent shunt revision.
